# Why do employees actively work overtime? The motivation of employees’ active overtime in China

**DOI:** 10.3389/fpsyg.2023.1120758

**Published:** 2023-04-24

**Authors:** Jinke Tan, Chunsheng Zhang, Zhengyang Li

**Affiliations:** ^1^School of Government, East China University of Political Science and Law, Shanghai, China; ^2^School of Business Administration, Shanghai University of International Business and Economics, Shanghai, China

**Keywords:** two-factor theory, initiative overtime work, factor analysis, data modeling, organizational environment, job attributes

## Abstract

**Introduction:**

Previous studies have defined “workaholic” effort as “bad effort” while work engagement is defined as “good effort.” Active overtime is a mapping of work effort, but at this stage there is still relatively little exploration of the motivation behind “good effort” in the Chinese context.

**Methods:**

This study explores the reasons that promote employees’ initiative to perform overtime work in Chinese enterprises based on the two-factor theory. The study mainly used data empirical research approaches, including exploratory factor analysis, validation factor analysis, and data modeling. The questionnaire scale was developed based on factors that have been proven to be of high reliability and validity. The data are mainly for employees who are currently employed in Chinese companies.

**Results and discussion:**

We received a total of 1741 valid questionnaires, which provided a good database for this study. The results of the study show that both motivational and hygiene factors can positively promote employees’ motivation to intentionally work overtime to a certain extent. Among them, overtime culture, institutional agreement, good physical office environment, career growth, financial rewards, and work challenges can positively promote motivation to work overtime. Work stress can increase the frequency and intensity of overtime work, but negatively promote motivation to work overtime. The study helps to improve enterprise management, optimize work design, and enhance psychological satisfaction.

## 1. Introduction

In 2022, there were many overwork deaths in China due to excessive overtime work, which once again generates attention and discussions among the public and academia. Excessive overtime increases the risk of death from stroke and ischemic heart disease. According to the 17-year survey data of 194 countries and regions around the world conducted by WHO and the ILO, overwork (more than 55 h per week) led to 745,000 deaths from stroke and ischemic heart disease in 2016 (Pega et al., 2021). Overtime work is not an old topic with the gradual reduction of the demographic dividend brought about by a large population base. A group of companies, represented by internet giants, have emerged and encourage extended working hours and employees to work overtime proactively. Thus, the “Neijuan” (Describing the act of voluntarily extending working hours to get paid for an existing job due to intense competition) and “996 pattern”(Describe a work status, start at 9:00 am and finish at 6:00 pm, work 6 days a week) have gradually evolved into a social norm. However, although encouraging extended working hours and advocating overtime work can promote higher economic benefits to a certain extent, the physical and mental health of employees cannot be guaranteed because of this. Extra working hours mean less time for rest and entertainment, and long-term high-load overtime work will also cause the health status of employees to decline (Dembe et al., 2005). This relates to the theme of presenteeism and the fact that workers intrinsically motivated to overwork for example go to work despite being sick, thus endangering their own health and that of their colleagues (Mazzetti et al., 2020). Companies and employees are in a state of confrontation regarding their overtime attitudes.

From the perspective of human capital, the greatest role of talented individuals is to create economic value, so how to maximize human capital is the most important thing for enterprise managers to consider (Wright and McMahan, 2011). Extending working hours has become an important factor for enterprises to improve business performance and this kind of expectation will be reflected in the human resource system of the enterprise (Brett and Stroh, 2003), it has a remarkable impact on employee motivation to work overtime. Employees tend to be consistent in the length of their working hours, for intensive overtime work is implicit in the management system, not engaging in overtime work instead would be defined as abnormal and out-of-group Most managers are often interested in promoting overwork among employees and establishing a reward system conducive to workaholic behavior (Clarke et al., 2015). Thus, workaholism represents socially accepted or even appreciated and rewarded obsessive–compulsive behavior in the organizational settings. In this situation, the length of overtime is even used as a basis for performance appraisal and evaluation of subordinate members (Leslie et al., 2012; Guo et al., 2020), overtime has thus evolved into a requirement that is implicit in the context of normal work, and employees were required actively or passively to accept overtime work.

Whether overtime work has a positive effect on corporate organizational performance is still controversial (Ko and Choi, 2018; Nguyen and Giang, 2020). However, the effect and impact of overtime on employees are relatively consistent, such as reducing life well-being; increasing psychological pressure (Rose, 1995); and causing sleep disorders, high blood pressure, and other diseases (Harma, 2006). Nishyama and Johnson's (1997) early research also found that excessive overtime behavior can lead to the death of employees due to overwork, and several facts regarding “death from overwork” incidents in China due to excessive overtime work have fully verified this conclusion. However, when overtime behavior comes from the subjective will of the employees, it can also have a positive effect on the employees themselves, such as enhancing their happiness (Beckers et al., 2007). Passive overtime work can lead to job burnout and even cause health hazards, but it is only established under the restriction of low salary levels. The group of employees who initiatively work overtime often has better income and working conditions (Beckers et al., 2008). Based on the research results of overtime work motivations, this study uses the two-factor theory to explore the factors that promote employees to initiatively work overtime aiming to provide some reference in improving work design, avoiding employment risks, and presenting new ideas in the study of overtime and overwork.

## 2. Literature review and research design

### 2.1. Two-factor theory and overtime-work

The two-factor theory, also known as the “Motivator-Hygiene Theory,” was first proposed by American behavioral scientist Frederick Herzberg (1959). This theory is an important basis of organizational behavior research, including two levels of satisfaction and dissatisfaction. Motivating factors are directly related to the work content itself, such as work achievements, organizational rewards, challenges of the work itself, and personal growth. Hygiene factors are directly related to the work environment, such as office environment, management policies, interpersonal relationships, guaranteed wages, and benefits. This theory denies the either-or-either relationship of motivational effects and points out that motivational factors and hygiene factors contain a dual relationship, that is, the opposite of satisfaction is no-satisfaction, the opposite of dissatisfaction is no-dissatisfaction, and reducing motivational factors will reduce job satisfaction, and reducing hygiene factors will not cause job dissatisfaction; increasing motivating factors will increase job satisfaction, and increasing health factors will not lead to changes in job satisfaction.

The two-factor theory provides a new idea for the study of organizational behavior, which includes the study of multi-employee overtime behavior. In terms of research on overtime behavior caused by work content itself, employee overtime behavior caused by work pressure might cause negative effects, resulting in job burnout and a sense of being controlled (Laurence et al., 2016), but if the overtime behavior caused by work pressure is viewed as a challenge, it can also improve employee motivation, satisfaction, and achievement (Mcallister et al., 2017). High achievement-oriented employees regard overtime as an opportunity for growth and are more likely to work overtime for expected growth. The challenges brought about by this growth orientation are an effective motivating factor (Bhargava and Pradhan, 2019), therefore, the attitudes of different types of employees in the face of the same incentives are also quite different.

In terms of employee overtime behavior caused by the organizational environment, common research involves the human resource management policy, compensation, and performance policy of the enterprise. The research conclusions involved are also quite different. For example, hygiene factors like employee training and performance management will evolve into a means for companies to exploit employees (Leslie et al., 2012), which will lead to a decline in employee job satisfaction (Van de Voorde et al., 2012). Additional economic remuneration can alleviate the negative impact of overtime to a certain extent and play an incentive role (Beckers et al., 2008).

Researchers have found that alternatives for traditional mentoring are positively related to career outcomes (Byrne et al., 2008). When individuals have strong job insecurity, in order to avoid losing their jobs, individuals will strive to perform better than their colleagues to demonstrate their value to the organization (Gilboa et al., 2008). The Job strain model proposed by Karasek shows that psychological problems at work are not caused by a single factor: Work demands are the result of joint action of job demands and job decision latitude (Karasek, 1979). Building on Deci and Ryan’s Self‐Determination Theory, Ilona van Beek examined the motivational correlates of workaholism, work engagement, and burnout, found that different types of motivational regulation are associated with different types of job‐related well‐being (Van Beek et al., 2012).

The two-factor theory has strong applicability and superiority in exploring the motivation of employees to work overtime initiatively. As far as the theoretical content itself is concerned, the motivational factors cover all kinds of factors that have been proven to cause employees to work overtime, such as the challenges of the work itself, the work responsibilities undertaken, the value of the work content to the enterprise, and the sense of accomplishment brought by the work results. Similarly, existing studies have also confirmed that health factors can affect employees’ overtime behavior, such as corporate policies, physical working environment, supervision, corporate culture, and salary. In addition, the responses of different individuals to the same factor are also quite different. Different types of employees have heterogeneity in their perceptions of workload, time preference, and other dimensions (Shepard and Clifton, 2000; Allen and Bunn, 2007), which lead to different Individual responses (Sturman and Walsh, 2014), provided the basis for the test of different demographic dimensions, and made this research feasible methodologically.

### 2.2. Theory and assumptions

#### 2.2.1. Organizational environmental factors and employee-initiated overtime work

##### 2.2.1.1. Corporate culture and employee-initiated overtime work

Corporate culture is a concentrated expression of organizational behavior and centripetal force. The procedure of shaping the cultural environment is a complex process of employee adaptation and knowledge transformation. This process will be affected by both the institutional environment and the physical office environment. Schein (2010) pointed out that corporate culture is formed in the process of continuous value input and behavioral code shaping, which occurs simultaneously with practical problem solving, and pointed out that the formation process of corporate culture includes leaders’ attention, response, strategy to key issues, and words and deeds; salary distribution standards for positions; and standards for talent selection, retention, dismissal. The formation of this cultural form originates from the founder’s value orientation, which is consolidated and strengthened by the implementation of the management, and finally shapes the cultural form of a certain enterprise through the flow of personnel and the recruitment of new employees (Feldman, 2002).

Because the formation of corporate culture is dependent on the founders and executives of the company, it is difficult to imitate and replace. Leaders shape a specific type of culture, often motivated by advocating employees’ active dedication and promoting the achievement of performance goals. Performance usually plays the role of an action guide, which can influence employees’ views on specific matters, thus forming a certain degree of behavioral norms, and ultimately promoting the improvement of organizational performance. Conversely, signals that improve organizational performance will also react to employee behavior, overtime culture is an important manifestation. Some scholars have pointed out that the organization will take an employee’s willingness to do overtime work into consideration to judge whether the employee has the potential for promotion (Perlow, 1996).

Obtaining high performance-oriented overtime has the potential to promote the emergence of workaholic tendencies to some extent (Ng et al., 2007), which is related to the so called controlled motivation (Van Beek et al., 2012), and might be influenced by both external norms and internal regulation, where external norms are not fully shared in the initial stages of introduction into the organizational environment (Deci and Ryan, 2000). In the absence of organizational recognition, employees work feverishly to meet these standards in order to achieve self-worth and recognition (Koestner and Losier, 2002), their perceptions of organizational environment are probable of contributing to employees’ workaholic tendencies (Porter, 2004), thus organizational factors play a significant role in employees’ maintenance of workaholic work status (Ng et al., 2007). Although employees with workaholic tendencies are primarily internally driven to work hard and to work overtime (Schaufeli et al., 2006), their willingness to work overtime may be caused by surrounding environment in which they are expected to work overtime, including consistent overtime behavior, expectations of upper-level managers, etc. Besides, an organizational climate that rewards work overtime might directly lead to employee overtime behavior. Thus, we put forward the following hypothesis:

*H1*: The company's overtime culture will positively promote employees' willingness to work overtime initiatively.

##### 2.2.1.2. Institutional environment and employee-initiated overtime work

As the early stage of cultural management, system management reflects the expected content of the enterprise and regulates the behavior of employees to a large extent, which includes the requirements for overtime. The management system of a company directly influences the organizational behavior of employees, who assign meaning to it based on their own understanding of the practices that take place within the company, this factor contributes to employees’ understanding of overtime behavior within the organization (Rousseau, 1988). As an integral basis for organizational management, internal management systems and procedures become key to providing a uniform explanation of behavior (Ostroff et al., 2003). Employees’ perception of the overtime atmosphere produces two results, one being an inherently positive attitude toward work, i.e., work engagement, the other is the intrinsically negative model, which is so-called workaholic (Schaufeli et al., 2009). A high level of work engagement is often closely associated with a positive work attitude, encompassing dynamism, risk-taking and absorption (Schaufeli et al., 2002), workaholism and work engagement are closely related to overtime, differences lie in that workaholism represents negative overtime while work engagement points to positive overtime. Employees with high levels of work engagement tend to be relatively more engaged and exhibit higher performance and job satisfaction (Schaufeli et al., 2008). Unlike overtime due to high work engagement, workaholic overtime causes higher levels of exhaustion (Taris et al., 2005), poorer social relationships outside the workplace (Schaufeli et al., 2008), and some degree of work–family conflict (Schaufeli et al., 2009). Although the motivating factors and resulting outcomes of overtime due to workaholism and work engagement are significantly different, their effects on employees’ work behavior performance are identical, that is, higher work intensity and longer overtime hours.

The human resource management system plays an uncritical role in guiding employees to work overtime and strengthening their normative constraints at the company level (Young and Choi 2018). Affected by the expected behavior habits and human resource management system, the employees of the company will maintain a consistent attitude towards overtime, for the reason that overtime behavior is implicit and advocated in the management system, not working overtime initiatively even becomes unacceptable and inappropriate (Cialdini et al., 1991), newly recruited employees will be motivated to work overtime because of the existence of this overtime atmosphere. Higher organizational performance is the result expected and pursued by the enterprise, human resource management systems set limits and benefits in the dimensions of employee personal development, compensation, benefits, job control, and employee relations to incentivize high-performance behavior and performance (Van de Voorde et al., 2012), employees have no choice but to accept this pattern (Brett and Stroh, 2003) to avoid the dilemma of being eliminated and marginalized. Based on this, this study proposes the following hypothesis:

*H2*: Institutional orientation positively promotes employees' willingness to work overtime initiatively.

##### 2.2.1.3. Physical office environment and employee-initiated overtime work

The research results of organizational behavior show that it is necessary to explore the motivation factors of employees working overtime from the perspective of the work environment (Sparks et al., 1997). The working environment serves as a significant hygiene factor, and a comfortable and safe office environment can be the premise and guarantee for employees to work effectively. The role of the physical office environment reflects both physical and psychological aspects, such as noise, heat, and ventilation, which can weaken overtime work’s impact on health. Comfortable office environments can promote the generation of positive emotions, enhancing the subjective experience of satisfaction, improving employees’ work engagement, and thus boosting the generation of innovative behaviors (Beckers et al., 2008). Correspondingly, a harsh or unfriendly work environment can increase employees’ work stress, cause workplace conflicts, and blur job roles (Jalagat, 2017). The research results based on the resource environment theory also show that a motivating work environment is an important part of a high-performance work system and has the property of enhancing employees’ work motivation. Its mechanism of action includes improving employees’ professional skills and promoting practice, enhancing their selfefficacy and selfconfidence to generate a sense of responsibility for competent work (White and Bryson, 2013). However, the physical office environment cannot be simply summarized as “good” or “bad,” especially in the context of the COVID-19 epidemic, many companies are trying to adopt flexible working system, which creates relatively better conditions for workers to control their schedule and choose working environment. However, flexible working seems to provide employees with more control over their work, but it actually creates a greater work-life imbalance. The use of smart and remote office equipment leads to employees being on call at all times and the work-life interface becomes blurred (Chung, 2022), which in turn also creates certain difficult factors for the study of overtime behavior. In order to optimize the research, we adopted the notion that physical office environment serves as a part of organizational climate, thinking that overtime behavior is influenced by employees’ motivational factors and personality traits on the one hand, and the organizational environment on the other (Ng et al., 2007; Fleck and Inceoglu, 2010). Their perceptions of observed environment and the meaning given to it might influence their behaviors (Schneider et al., 2013). Organizational climate which encourage employees to work overtime is probable of increasing their motivation to work overtime voluntarily to a greater extent, and they are more likely to show intrinsic compulsion and internal drive to work in a high-intensity organizational environmental climate (Johnstone and Johnston, 2005), with the phenomenon of workplace overtime having been defined as an excessive work environment (Mazzetti et al., 2014). The physical work environment, as a critical component of the organizational environment, can directly influence employee behavior and perceptions of work,further has a potential impact on employee motivation to work overtime. Based on previous research results, we believe that although the physical office environment is a hygiene factor, a higher level of comfort in the office environment can positively promote employees’ feelings, thereby affecting overtime work behavior. For this reason, this study proposes the following hypothesis:

*H3*: A good physical office environment can positively affect employees' motivation to work overtime initiatively.

#### 2.2.2. Work attributes and employee-initiated overtime work

According to the theory of dual factors, this study focuses on the work content, work breadth and depth, and analyzes the direct and indirect impact of employees based on work attribute factors, such as obtaining career growth, promotion of professional grade, increased economic income, and performance improvement.

##### 2.2.2.1. Occupational growth and employee-initiated overtime work

Pressure trading theory attributes the role of individual and objective situations to the root cause of pressure, suggesting that work-related pressure has the potential to generate positive psychological motivation (Lazarus and Folkman, 1984). Positive experience, such as higher work quality requirements and shorter time cycles, is a favorable factor in promoting career development for individuals, which can increase professional satisfaction (Cavanaugh et al., 2000). Besides, employees view pressure from work that promotes personal competence and is aligned with their career direction as a challenging pressure, under such circumstances they actively choose the best strategies and practices to achieve higher work goals, thus show higher motivation and innovation (Boswell et al., 2004). Therefore, we speculate that when the direction of career development is consistent with the occupation they are engaged, employees are more likely to show the subjective initiative of work. Based on this, the following hypothesis is proposed:

*H4*: High career growth orientation will play a positive role in promoting the initiative overtime work motivation of employees.

##### 2.2.2.2. Economic remuneration and employee-initiated overtime work

Obtaining economic remuneration is one of the important motivations for employees to work overtime, higher overtime remuneration can weaken the negative effects of long-term overtime work, but when employees notice that the economic remuneration obtained is less than the deserved return, they will have dissatisfaction (Siegrist, 1996). Economic remuneration covers the passive willingness of workers to work overtime to a certain extent, creating an illusion of being initiatively working overtime. Related research results show that behaviors aiming to obtain economic overtime-remuneration function as an incentive role only in the situation that the goal of overtime work is consistent with that of their career plan (Sonnentag et al., 2018). When completing the task of work can bring high performance, a high position, or a better salary, employees’ overtime mobility will be further improved (Scott et al., 2010). However, research has also fully confirmed that in terms of promoting active overtime, the incentives of economic income to high-income groups are relatively weak (Beckers et al., 2008). It is not difficult to find that active overtime acts because of the return of remuneration are only established in a relatively limited assumption situation. Under the pressure of economic downward, employees will work overtime because they are worried about unemployment, thus showing an illusion of initiative overtime work. Based on this, we propose the following hypothesis:

*H5*: Economic factors have a positive role in promoting the motivation for initiative overtime working. Employees with low income and relatively clear career planning are more likely to work overtime due to economic remuneration.

##### 2.2.2.3. Working load and employee-initiated overtime work

Heavy workload is one of the major contributing factors to employees’ overtime work, usually related to short working hours, heavy workloads, and high work requirements. As far as employees are concerned, overtime work caused by workload can be regarded as poor performance (Nguyen and Giang, 2020), making them feel controlled and emotionally negative (Laurence et al., 2016), and thus even leading to burnout (Ko and Choi, 2018) and causing a sense of deprivation (Chen et al., 2020).

To increase *per capita* output, extending working hours is a common practice in many companies. This expectation and the associated human resource policy will form an implicit constraint (Barnes et al., 2016), as a result of which, the employee’s choice of improving work efficiency and actively working overtime becomes blurred, forming an illusion of being actively working overtime but in fact, the frequency of overtime has no actual relationship with motivation, for this reason, we propose the following hypothesis:

*H6*: Workload negatively promotes employees' motivation to work overtime initiatively.

##### 2.2.2.4. Work challenges and employee-initiated overtime work

The Job Demand-Resource Model (JD-R) provides a reasonable explanation for the combination of work outcomes and resources, arguing that work output is an organic combination of personal resources and work resources, and is in a state of constant change over time (Taris and Schaufeli, 2015). When an individual’s judgment of the value of their work is positive, they will be more invested and their job satisfaction will also increase (Hackman and Oldham, 1976). Accordingly, when faced with working overtime, they will make subjective judgments on the need for overtime according to the situation, thinking about whether the effort can bring about ideal results, including situational factors such as personal development and career achievement (Lepine et al., 2016). When employees see overtime as a work challenge, they are more engaged and enthusiastic (Mcallister et al., 2017).

The propensity to work overtime due to high job challenge also has a strong correlation with employee personality traits. Basic factors that lead to workaholism include obsessive–compulsive traits, achievement orientation, perfectionism, and responsibility (Ng et al., 2007; Liang and Chu, 2009).

Another concept corresponding to workaholism is dedication, the difference is that workaholics are influenced and driven by external controlled factors, they tend to devote more energy and time to achieve better results (Mazzetti et al., 2018). Employees with workaholic tendencies are more susceptible to intrinsic personality traits, they are more likely to be driven by extraordinary goals of achieving self-enhancement that exist in recognized self-worth and social standards (Van Beek et al., 2012), as well as the tendency to engage in unsolicited work is more pronounced when compulsive work drives them to high levels of commitment (Mazzetti et al., 2020). Therefore, we speculate that when employees regard work as a challenge, they are more likely to work overtime because of the high challenge of the work:

*H7*: High job challenge positively promotes employees' motivation to work overtime initiatively.

### 2.3. Research design

**Figure 1:**
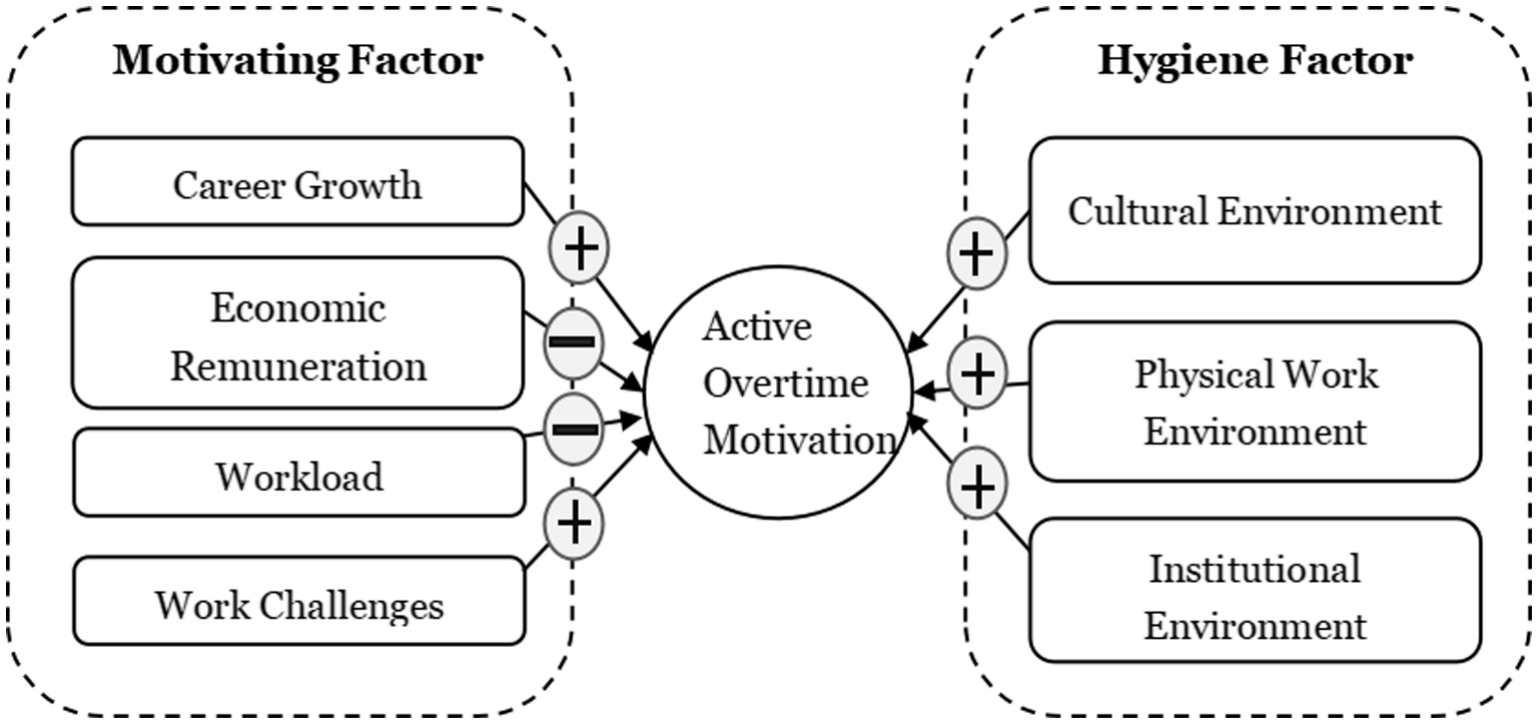
Research model.

The procedures of this study mainly involve model designing, questionnaire designing, data collection and empirical analysis. In the theoretical model design session, based on two-factor theory, we systematically analyzed and categorized the motivation factors of employees working overtime and used the empirical research method of data analysis. The core purpose of this paper is to explore and analyze the incentives for employees to actively work overtime, research model is shown in Figure 1.

## 3. Research methods

### 3.1. Questionnaire design

Based on the variables of each dimension that have been empirically studied, demographically, gender, age, education level, marital status, monthly pre-tax income, monthly consumption as a proportion of income, nature of business/unit, size of business/unit, job category, and job level were selected as the study grouping variables, and the variable assignments are shown in Table 1.

**Table 1:** Demographic variables and assigned values.

Variable	Variable assignment
Gender	“1” = Male; “2” = Female
Age	“1” = under 20 years old; “2” = 21–25 years old; “3” = 26–30 years old; “4 “= 31–35 years old; “5″ = 36–40 years old; “6″ = 41–45 years old; “7″ = 46–50 years old; “8″ = 51–55 years old; “9″ = 55 years old and above
Education	“1” = specialist and below; “2” = bachelor; “3” = master; “4” = doctor
Marital status	“1” = unmarried; “2” = married with no children; “3” = married with children
Monthly income before tax	“1” = less than $6,000; “2” = $6,000-10,000; “3” = $10,000-15,000; “4” = 15,000–20,000 yuan; “5” = 20,000–30,000 yuan; “6” = 30,000–50,000 yuan; “7” = $50,000 or more
Monthly expenses to income ratio	“1” = less than 20%; “2” = 20–40%; “3” = 40–60%; “4 “= 60–80%; “5″ = more than 80%
Enterprise size	“1” = less than 100 people; “2” = 100–300 people; “3” = 300–1,000 people; “4 “= 1,000–2000 people; “5″ = more than 2000 people
Job category	“1” = Technology/R&D category; “2” = Management/Administration category; “3” = Marketing category; “4 “= Teacher/Consultant/Consulting; “5″ = Professional; “6″ = Skilled worker; “7″ = Other
Position level	“1” = general staff; “2” = junior manager (supervisor, manager and related position); “3” = middle manager (directors and related positions); “4” = Senior managers

The latent variables included overtime due to the organizational environment and overtime due to the work itself, with a total of 22 items. Among them, 10 questions were about work environment factors; 12 questions were about work itself factors, and all the tested variables were positively scored using a 5-point Likert scale, with a score of 1 indicating the lowest degree and 5 indicating the highest degree.

The scale was developed based on factors that have been empirically validated by scholars with high reliability and validity, including the career growth test scale. The career growth test scale was developed based on the results of a study (Carmeli and Gefen, 2005) on horizontal and vertical job mobility, and three factors were selected: career knowledge base, job advancement, and career aspirations. The economic compensation test scale was based on the research results of Aryee et al. (1996) and Valcour and Ladge (2008), including three factors: pay level, overtime allowance, and indirect benefits.

The Job Challenge Test Scale (JCTS) refers to Lepine’s (2016) Measuring Job Challenge Scale, and based on the concept of challenging work core, we selected three factors for scale development: job challenge, outcome delivery, and motivating potential. Working load scale is based on the concept of job function enlargement and enrichment proposed by Robbins (2004), combined with work time pressure, we selected three factors—namely work module, task requirements, and time constraints—as the basis for scale development. In terms of cultural environment, based on the concept of corporate culture evolution path proposed by John and Alla (2006), and the consistent behavioral evolution path proposed by Schein (2010) and Feldman (2002), we selected management, direct leaders, colleagues in the same department, plus overtime atmosphere as the basis for the development of the cultural environment test scale. In addition, drawing on Beckers et al. (2008), we set the degree of willingness to work overtime and income level as observational variables. Chang et al. (2021) showed that job autonomy was effective in reducing emotional exhaustion caused by overtime work, combined with the clue that overtime work has the potential to cause emotional exhaustion, we set job autonomy as the study-observed variable. There is a certain degree of correlation between the direction of career planning, an individual’s major, and the job they are working in. A high degree of clear career planning and growth orientation can positively promote career focus, so this research questionnaire sets the correlation between career planning and major as the observation variable.

### 3.2. Data collection

The data collection of this study consisted of two parts: pre-survey and formal survey. During the pre-research phase, we effectively gathered 108 valid questionnaires from February to April 2022. We tested the reliability and validity of the data using SPSS software, further revised and deleted the ambiguous items that affected the reliability and validity of the data. The second phase of formal data collection was conducted from May to October 2022, in this stage, 2,000 questionnaires were distributed, 1,826 questionnaires were collected, 85 invalid questionnaires were excluded, and 1,741 valid questionnaires were finally included in the data analysis (see Table 2 for details).

**Table 2:** Detailed data collection results.

Dimension	Sub-dimension	Data	Dimension	Sub-dimension	Data
Gender	Male	977	Education	Specialist and below	407
	Female	764		Bachelor	768
Age	<21	170		Master	378
	21–25	318		Doctor	188
	26–30	573	Marital Status	Unmarried	416
	31–35	387		Married with no children	644
	36–40	187		Married with children	681
	41–45	48	Job Category	Technology/R&D	364
	51–55	58		Management/Administration	386
Position level	General Staff	677		Marketing	330
	Junior Manager	891		Teacher/Consultant/Consulting	288
	Middle Manager	143		Professional	252
	Senior Manager	30		Skilled worker	121

### 3.3. Data analysis and hypothesis test

#### 3.3.1. Test scale reliability validity analysis

The reliability analysis of the test scale using SPSS25 in this study showed that the Cronbach’s alpha value for each dimension of the test variables was higher than 0.8, showing that the overall reliability was at a high level. Exploratory factor analysis was applied to each dimension of each test dimension, and the results showed that the KMO values of each variable were higher than 0.7, and Bartlett’s sphericity test showed that the significance level of each variable was 0.000, which was more suitable for the method of factor analysis. The reliability analysis of the test scale (also using SPSS25) showed that the Cronbach’s alpha value for each dimension of the test variables was higher than 0.8, showing that the overall reliability was at a high level. Exploratory factor analysis was applied to each dimension of each test dimension, and the results showed that the KMO values of each variable were higher than 0.7. Bartlett’s sphericity test showed that the significance level of each variable was 0.000, which was more suitable for the method of factor analysis.

The results of the factor analysis showed that the cumulative variance explained by the three factors included in the work environment variable, cultural environment (CE), physical environment (PE), and institutional environment (IE), were all higher than 71%. The cumulative variance explained by the four factors of career growth (CG), economic remuneration (ER), workload (WL), and work challenge (WC) included in the job attribute variables were all higher than 89%. All factor loadings were higher than 0.9, indicating that the factors had relatively desirable validity (see Table 3).

**Table 3:** Reliability and validity analysis (*N* = 1741).

Variable	Dimension	Title	Cronbach’s α	KMO	Factor loading	Bartlett’s test of sphericity	Cumulative variance
Work environment factors	Cultural Environment (CE)	CE_1	0.915	0.815	0.898	0.000	79.756%
		CE_2			0.905		
		CE_3			0.904		
		CE_4			0.864		
	Physical Environment (PE)	PE_1	0.809	0.771	0.851	0.000	71.373%
		PE_2			0.846		
		PE_3			0.838		
	Institutional Environment (IE)	IE_1	0.824	0.719	0.846	0.000	73.982%
		IE_2			0.864		
		IE_3			0.870		
Job attribute factors	Career Growth (CG)	CG_1	0.944	0.774	0.951	0.000	88.082%
		CG_2			0.947		
		CG_3			0.949		
	Economic Remuneration (ER)	ER_1	0.897	0.775	0.948	0.000	90.066%
		ER_2			0.952		
		ER_3			0.947		
	Workload (WL)	WL_1	0.812	0.713	0.843	0.000	72.911%
		WL_2			0.872		
		WL_3			0.847		
	Work Challenge (WC)	WC_1	0.940	0.774	0.952	0.000	89.855%
		WC_3			0.951		
		WC_3			0.942		

#### 3.3.2. Validation factor analysis

**Figure 2:**
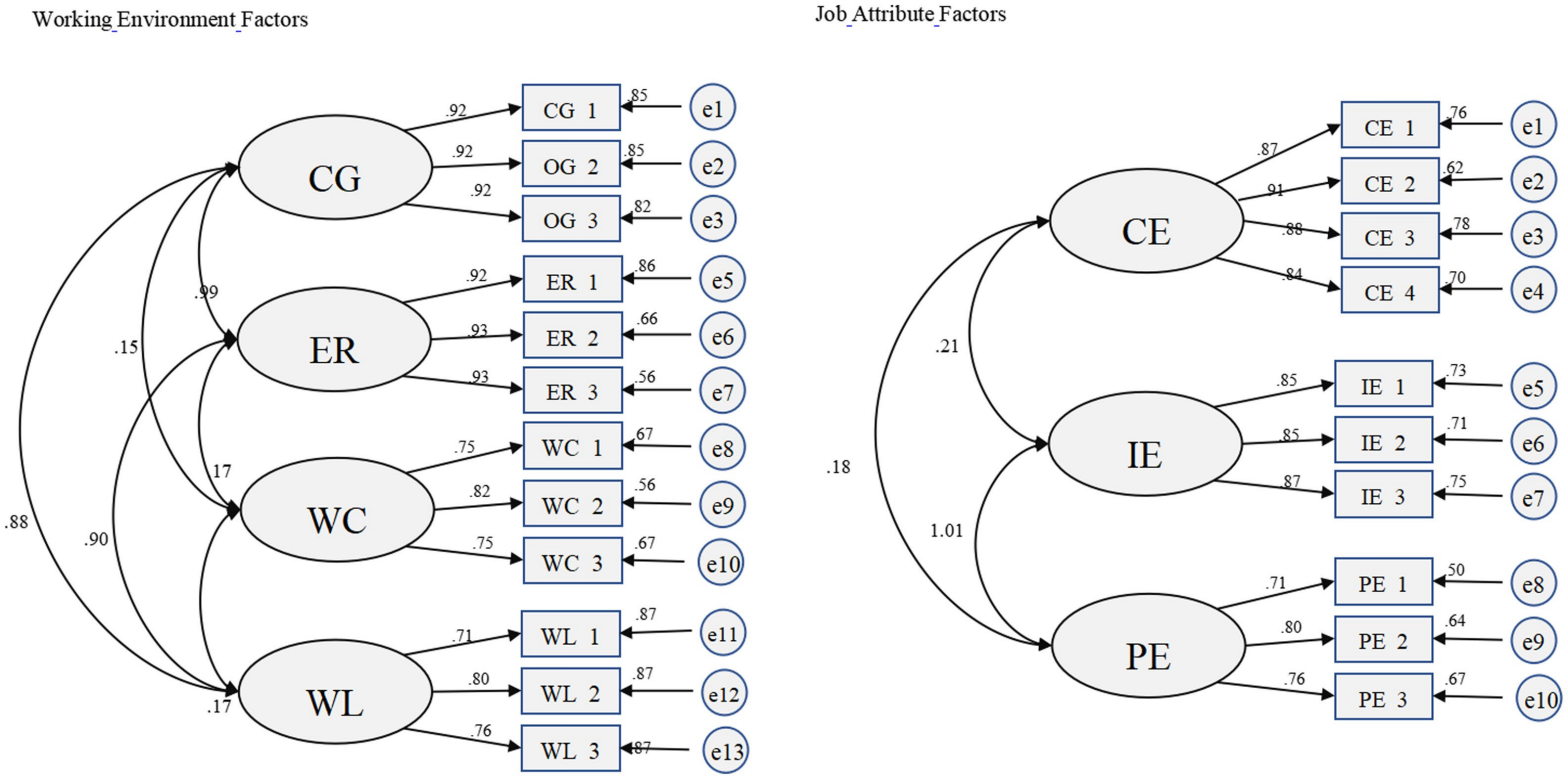
Standardization of each factor of overtime work.

To ensure that the study model had high credibility, AMOS 25.0 was used for model validation of the data to ensure that the study mold had high structural, combined, and discriminant validity. The results of the analysis showed that the values of CMIN/DF for both environmental and work factors were between 1 and 2, RESEA values were less than 0.005, GFI, CFI, AGFI, TLI, and CFI values were higher than 0.9, and the fit of both models was high (see Table 4 for indicator values and Figure 2 for standardized solutions).

**Table 4:** Validation factor analysis.

Dimension	CMIN/DF	GFI	RMSEA	AGFI	NFI	TLI	CFI
Environmental factors1	1.975	0.984	0.037	0.972	0.932	0.950	0.965
Environmental factors2	2.278	1.000	0.356	0.230	1.000	0.000	1.000
Job attribute factors1	1.144	0.955	0.009	0.991	0.998	1.000	1.000
Job attribute factors2	2.808	1.000	0.450	0.162	1.000	0.000	1.000
Reference standards	<3	>0.9	<0.08	>0.8	>0.9	>0.9	>0.9

“1” represents no potential impact factor added; “2” represents potential impact factor has been added.

#### 3.3.3. Common method bias testing

To reduce the impact of common method bias on the study results, we designed and processed the study in two dimensions: procedural and statistical methods. For the study procedure, we used a pre-research data collection method to collect respondents’ feedback and systematically modify the research questionnaire in order to minimize the effects of ambiguity and cognitive dissonance. In addition, to reduce the effects caused by time and space, we collected data centrally, eliminated questionnaires with too long and too short response times, tested respondents’ concentration in setting repeated question items, and removed data with poor concentration.

At the level of statistical methods, we conducted exploratory factor analysis without rotation on the data involved in the questionnaire using Harman’s single-factor test, and a total of four factors with eigenvalues greater than 1 were extracted, of which the variance explained by the first factor was 42.856%, referring to (Podsakoff and Organ, 1986), the variance explained by less than 50% can be considered not There is a serious common method bias (see Table 5).

**Table 5:** Factor analysis to verify common method bias.

Total variance explained						
Item	Initial Eigenvalues			Extraction of the sum of squares of loads		
	Sum	Percentage of Variance	Accumulation %	Total	Percentage of variance	Accumulation %
1	9.428	42.856	42.856	9.428	42.856	42.856
2	3.422	15.553	58.409	3.422	15.553	58.409
3	2.845	12.932	71.341	2.845	12.932	71.341
4	1.056	4.801	76.142	1.056	4.801	76.142
5	0.587	2.668	78.810			
6	0.514	2.337	81.147			
7	0.477	2.168	83.316			
8	0.459	2.088	85.404			
9	0.437	1.988	87.392			
10	0.406	1.846	89.238			
11	0.386	1.755	90.993			
12	0.355	1.613	92.605			
13	0.316	1.438	94.043			
14	0.176	0.801	94.844			
15	0.160	0.727	95.571			
16	0.155	0.703	96.274			
17	0.150	0.683	96.957			
18	0.145	0.658	97.615			
19	0.140	0.637	98.252			
20	0.134	0.608	98.860			
21	0.128	0.580	99.440			
22	0.123	0.560	100.000			

Extraction method: principal component analysis.

To further analyze the common method bias, we used the suggestion of Podsakoff et al. (2003) to introduce an unmeasured potential common factor into the original model, using the Amos24 software to test the model for common method bias and determine the severity of the common method bias by observing the change in the degree of fit of the model. The validation results showed that the fit of the model became unsatisfactory after the addition of the potential factor (see Table 4), so it can be presumed that the common method bias of this study is in a relatively desirable range.

#### 3.3.4. Correlation of variables

Correlation analysis of the study’s latent variables using SPSS25 showed varying degrees of correlation among the variables, including the dimensional demographic variables of gender, age, education level, and marital status. The dimensions of income level, consumption share, company type, company size, career plan, professional field, position type, position level, overtime frequency, overtime hours, degree of job control, and degree of willingness to work overtime also showed strong correlations, which laid a better foundation for establishing data model analysis (see Table 6 for correlations of observed variables).

**Table 6:** Correlation analysis of variables.

	M	SD	1	2	3	4	5	6	7	8	9	10	11	12	13	CE	PE	IE	CG	ER	WC	WL
Gender	1.44	0.50	1																			
Age	3.27	1.39	0.084**	1																		
Education	2.20	0.92	0.007	0.379**	1																	
Marriage	2.15	0.78	−0.191**	0.469**	0.174**	1																
Income	3.55	1.61	−0.336**	0.506**	0.411**	0.382**	1															
Expenditure	3.07	1.19	0.01	−0.431**	−0.240**	−0.293**	−0.407**	1														
Scale	2.76	1.20	−0.129**	0.150**	−0.059*	0.218**	0.132**	−0.134**	1													
Major relation	3.42	1.23	0.031	0.429**	0.397**	0.327**	0.457**	−0.404**	0.148**	1												
Position lever	1.74	0.69	−0.076**	0.764**	0.360**	0.455**	0.537**	−0.408**	0.143**	0.442**	1											
Self-determination	3.14	1.21	−0.061*	0.482**	0.232**	0.463**	0.440**	−0.366**	0.169**	0.384**	0.470**	1										
Overtime frequency	2.09	0.82	−0.233**	0.186**	0.041	0.266**	0.255**	−0.100**	0.221**	0.192**	0.201**	0.211**	1									
Overtime hours	2.14	0.75	−0.255**	0.321**	0.213**	0.300**	0.383**	−0.190**	0.119**	0.263**	0.333**	0.280**	0.222**	1								
Overtime voluntary	3.13	1.21	−0.255**	0.497**	0.409**	0.423**	0.539**	−0.389**	0.132**	0.441**	0.505**	0.414**	0.240**	0.364**	1							
CE	3.16	1.01	−0.177**	0.493**	0.445**	0.371**	0.416**	−0.323**	0.016	0.344**	0.470**	0.372**	0.087**	0.274**	0.622**	1						
PE	3.33	1.13	−0.113**	0.262**	0.061*	0.257**	0.164**	−0.048*	0.04	0.031	0.239**	0.208**	0.152**	0.127**	0.301**	0.210**	1					
IE	3.37	1.03	−0.03	0.231**	0.107**	0.181**	0.173**	−0.139**	0.021	0.132**	0.229**	0.153**	0.121**	0.099**	0.297**	0.202**	0.742**	1				
CG	3.04	1.24	−0.440**	0.379**	0.279**	0.381**	0.477**	−0.324**	0.206**	0.342**	0.401**	0.318**	0.318**	0.349**	0.897**	0.506**	0.243**	0.223**	1			
ER	3.06	1.26	−0.443**	0.389**	0.315**	0.393**	0.487**	−0.306**	0.179**	0.352**	0.416**	0.315**	0.320**	0.345**	0.906**	0.528**	0.267**	0.239**	0.941**	1		
WC	3.01	1.30	−0.386**	0.391**	0.455**	0.380**	0.597**	−0.332**	0.106**	0.424**	0.434**	0.375**	0.201**	0.374**	0.908**	0.573**	0.186**	0.160**	0.838**	0.856**	1	
WL	2.03	0.98	−0.197**	0.213**	0.300**	0.163**	0.181**	−0.086**	−0.03	0.089**	0.208**	0.152**	0.014	0.102**	0.123**	0.501**	0.048*	0.024	0.134**	0.149**	0.158**	1

* Indicates significant correlation at the 0.05 level (two-tailed); ** Indicates significant correlation at the 0.01 level (two-tailed).

## 4. Research results

### 4.1. Hypothesis testing

Based on the results of the correlation analysis test, regression analysis was conducted with the help of SPSS25 for each dimension of latent variables that showed a significant correlation to test the hypotheses. The results of the regression analysis showed that the overtime culture of enterprises had a positive correlation with the willingness to work overtime initiatively, and Hypothesis 1 was verified. Hypothesis 2 was tested for the positive contribution of a physical office environment to incentivize overtime. The institutional environment positively contributes to motivation to work overtime initiatively, thus Hypothesis 3 is tested. Career growth positively promotes initiative overtime work motivation, and Hypothesis 4 was verified. Workload negatively contributes to initiatively overtime work motivation, Hypothesis 6 was verified. Work challenge positively contributes to motivation to work overtime initiatively, and Hypothesis 7 was verified (see Table 7 for the regression model of the above findings). To further verify the influence of organizational environment and work factors on overtime behavior, regression models were established with each factor as the outcome variable, and the results are shown in Table 8.

**Table 7:** Model regression analysis 1.

Variable	Model I	Model II	Model III	Model IV	Model V	Model VI	Model VII	Model VIII
(Constants)	1.469***	1.953***	2.072***	2.072***	1.898***	1.977***	2.433***	2.444***
Income	0.069***	0.077***	0.076***	0.074***	0.06***	0.059***	−0.001	−0.002
Major relation	0.162***	0.127***	0.124***	0.128***	0.026***	0.019**	0.001	
Position lever	0.114*	0.074	0.059	0.054	0.08***	0.071***	0.064***	0.064***
Marriage	0.15***	0.1**	0.067**	0.073*	0.026*	0.017	0.014	0.016
Education	0.183***	0.068**	0.076**	0.074***	0.073***	0.051***	−0.025**	−0.008
Gender	−0.443*	−0.3***	−0.276***	−0.288***	0.41***	0.463***	0.431***	0.395***
Expenditure	−0.105***	−0.08***	−0.093***	−0.086***	−0.01	−0.019**	−0.023***	−0.021***
Career plan	0.102***	0.095***	0.112***	0.105***	0.026**	0.018*	0.003	0.001
Age	0.119***	0.049**	0.025*	0.029	−0.039***	−0.039***	0.004	0.007
Overtime work Hours	0.096**	0.093**	0.092***	0.093***	0.028*	0.033**	0.017	0.014
CE		0.406***	0.39***	0.387***	0.176***	0.153***	0.102***	0.142***
PE			0.17***	0.101***	0.063***	0.048***	0.039***	0.038***
IE				0.091***	0.031**	0.028**	0.065***	0.062***
OG					0.955***	0.492***	0.345***	0.343***
ER						0.536***	0.306***	0.303***
WC							0.525***	0.506***
WL								−0.071***
*R* ^2^	0.483	0.551	0.569	0.571	0.887	0.908	0.942	0.945
△*R*^2^	0.003	0.068	0.017	0.002	0.316	0.021	0.034	0.002
*F*	161.882***	193.227***	189.821***	176.836***	969.697***	1135.686***	1758.968***	1725.936***

**Table 8:** Model regression analysis 2.

Variable	Organizational Environment			Job Attributes			
	CE	PE	IE	CG	ER	WC	WL
(Constants)	−1.386***	−1.216***	−1.01***	−1.46***	−1.599***	−2.030***	0.229
Gender	−0.261***	−0.071	0.105*	−0.449***	−0.461***	−0.285***	−0.584***
Age	0.14***	0.124***	0.048*	0.03***	0.028***	−0.061***	0.142***
Education	0.202***	−0.062**	−0.032	−0.054***	−0.014	0.106***	0.329***
Marriage	0.083**	0.146***	0.059	−0.027*	−0.004	−0.006	0.077**
Income	−0.046**	−0.02	0.006	−0.037***	−0.037***	0.075***	−0.048**
Expenditure	−0.023	0.093***		−0.019**	0.007	0.023***	0.005
Scale	−0.065***	−0.032*	−0.032	0.049***	0.028***	−0.006	−0.05**
Career plan	−0.004	−0.123***	−0.022	−0.002	0.004	0.024***	−0.03
Major relation	0.031*	−0.019	−0.063**	0.008	0.011	0.027***	0.011
Position lever	0.05	0.063	0.105*	−0.069***	−0.044**	−0.048**	0.047
Self-determination	0.051***	0.062***	−0.001	−0.041***	−0.05***	0.004	0.054**
Overtime frequency	−0.108***	0.074**	0.072**	0.087***	0.092***	−0.062***	−0.068**
Overtime hours	−0.015	−0.025	−0.03	−0.008	−0.025**	0.015	−0.046
Overtime voluntary	0.326***	0.22***	0.23***	0.735***	0.731***	0.674***	−0.126***
*R* ^2^	0.49	0.167	0.112	0.871	0.881	0.878	0.179
△*R*^2^	0.49	0.167	0.112	0.871	0.881	0.878	0.179
*F*	118.395***	26.611***	15.550**	834.960***	917.000**	889.374***	26.944***

Motivation of overtime behavior caused by organizational environment factors and job factors are outcome variables, where * indicates *p* < 0.1, ** indicates *p* < 0.05, *** indicates *p* < 0.001.

### 4.2. Analysis of results

Overall, the degree of employee willingness to work overtime initially is influenced by both work environment factors and the work attributes. The degree of influence varies considerably with the characteristics of demographic variables, including gender, age, education level, and marital status. The degree of willingness to actively work overtime is also impacted by the current status of the employee and the company, including income and expense levels, company size, area of expertise, job level, self-determination of work, and the intensity/frequency of overtime work.

#### 4.2.1. Organizational environment enhances motivation to overtime to varying degrees

Organizational environment, as an essential hygiene factor, provides an important basis for research and analysis of work efficiency improvement, work resource allocation, and organizational performance output. Combined with the results of this study, we found that overtime culture, management system, and physical office environment as hygiene factors can positively promote employees’ motivation to work overtime. The characteristics of the job itself, as part of job design and functional design, are often categorized as motivational factors. For the results of this study, challenging task design, career growth, and higher remuneration can all positively contribute to overtime work motivation, while heavier workloads negatively contribute to the subjective willingness to work overtime.

#### 4.2.2. Job attributes enhances motivation to overtime

There is also clear variability in the degree to which the attributes of the job itself promote voluntary overtime work for different types of employees. Career growth orientation positively promotes motivation to work overtime among male and older employees, and negatively promotes motivation to work overtime among employees with higher education levels and higher incomes. Obtaining financial income positively promotes overtime behavior among male and older employees, and the willingness to work overtime due to obtaining financial income decreases continuously as income levels increase. Motivation to work overtime due to work challenge gradually decreases with age and income level, and male employees are more motivated to work overtime due to high work challenges than females. High workload increases the frequency of overtime work for employees to a greater extent, these empirical results show that male, high age, high education level, and married (childbearing) employees have a stronger motivation to work overtime voluntarily due to workload. However, this willingness to work overtime voluntarily due to a high workload will gradually diminish as income increases.

#### 4.2.3. Differences in the motivations for overtime among divergent groups

Employees of different demographic dimensions also showed a large variability in voluntary overtime willingness. Specifically, (1) female employees show higher motivation to work overtime compared to male employees; (2) willingness to work overtime initiatively shows a gradual increase with age, but this correlation becomes insignificant after work challenge and workload factors are included in the model; (3) income level has a more limited and obvious positive contribution in promoting overtime work intentions, but the willingness to work overtime voluntarily decreases with the increase of consumption level; (4) the correlation between major and career, career plan and current career can positively promote overtime motivation to a certain extent, however this correlation becomes insignificant after the factors of work challenge and work pressure are included in the model; (5) the willingness to work overtime tends to increase with the increase of position level; (6) marriage and childbearing status can promote overtime motivation to a limited extent, but the significance gradually decreases with the inclusion of financial reward, job challenge, and job stress factors into the model.

Further analysis shows that although female employees are less likely to work overtime initiatively due to overtime culture, career growth, financial reward, work challenge, and work pressure factors compared to male employees, they are more likely to work overtime due to the orientation of the overtime institution. The willingness of employees to work overtime due to the influence of overtime culture, management system, and physical office environment gradually increases with age, and older employees are more likely to work overtime voluntarily due to career growth, financial rewards, and workload factors, but less likely to work overtime voluntarily due to the influence of work challenges.

The improvement of education level can promote the degree of adaptation to overtime culture, further employees with higher education levels are more likely to voluntarily work overtime due to high work challenges compared with those with lower education levels. However, higher education level also reduces the subjective compliance with the management system oriented to overtime. Furthermore, willingness to work overtime due to career growth gradually decreases with the increase in education level. Moreover, employees with different statistical variables showed more significant variability in the extent to which they were motivated to work overtime by organizational environmental factors. In terms of cultural environment, female and high-income employees show more tendency to resist the company’s overtime culture, and employees that are older, highly educated, and married (childbearing) are more likely to work overtime initiatively due to the existence of overtime culture. The physical office environment also serves as boosting factor to some degree, and the tendency to work overtime due to a good physical office environment gradually strengthens as age increases and marital status changes (single to married/childbearing). Institutional orientation has a greater impact on men and older employees than on women and younger employees.

#### 4.2.4. Findings for other dimensions

Our study also revealed that the extent to which employees are influenced to work overtime by the overtime culture and workload diminishes as the company grows in size. Clear career planning has no significant effect on employees’ adaptation to the overtime culture and system, but it weakens the influence of the office environment and positively promotes the behavior of working overtime due to workload. The degree of relevance of majors to current occupation positively promotes the active adaptation to overtime culture and the behavior of overtime work due to work challenges. The behavior of overtime work due to institution orientation is strengthened, while the motivation for overtime work due to career growth, income, and work challenges is weakened as the position level increases. Higher levels of self-determination of work promote active adaptation to the overtime culture and increase the willingness to work overtime due to a good physical office environment, but at the same time, as the level of job self-determination increases, the willingness to work overtime due to career growth and economic income decreases, and the willingness to work overtime due to high workload is more tolerable. The higher frequency of overtime does not promote the behavior of overtime due to the existence of overtime culture and higher work challenges/workload but positively promotes overtime work intention due to the physical work environment, institutional norms, career growth, and financial rewards.

### 4.3. Cause analysis

#### 4.3.1. Organizational environment and initiatively overtime work behavior

The results of this study found that hygiene factors can motivate employees to perform overtime work voluntarily to some extent, like overtime culture, management system oriented to overtime work, and a good physical office environment. Drawing on the characteristics of the organizational environment, we focus on the interpretation of the results from the perspective of corporate culture, referring to Schein's (2010) definition, which considers corporate culture as the values, beliefs, or ideas shared by employees within the organization. Corporate culture, as an important symbol that distinguishes the characteristics and values of the firm, directly influences the behaviors and attitudes of employees and ultimately affects organizational performance (Hogan and Coote, 2014). It can be assumed that the role of corporate culture contains both guiding and constraining dimensions, its influence on employees’ behavior is lasting and more subtle. In the case of employees themselves, their level of adaptation to a given corporate culture will be able to determine whether they will stay in the company for a long time to create value, precisely, the results and level of adaptation to the culture of employees who start to adapt to the new environment from the beginning of their employment may affect their behavior and performance (Federici et al., 2019). The adaptation level of overtime culture determines its ability to keep up with the overall work pace, a process that Safavi and Bouzari (2019) define as employees’ adaptability to new environments, one of their core competencies, and the increasing importance of this ability in performance appraisal (Kang and Lee, 2021), so it is reasonable to believe that overtime culture in which leaders and colleagues of overtime habits can contribute to individuals’ motivation to work overtime.

#### 4.3.2. Work factors and overtime behavior

Another significant element of this study is to examine the contribution of the attributes of the job itself to plus active overtime motivation. It is found that high growth-oriented, high economic reward-oriented, and high challenge jobs can all positively contribute to overtime motivation, and larger workloads can increase the frequency of overtime work but play a negative role in promoting employees’ motivation to work overtime. Based on the definition of the two-factor theory, we attempt to elaborate a reasonable explanation from the following aspects.

Career growth in a given organization consists of both organizational and professional identity, i.e., skills and knowledge in the field of expertise that are valuable in the organization in which they are employed and have an upward trajectory as the organization grows. In the context of this study, we believe that career growth is the product of the interaction between organizational and professional identities, including job satisfaction, organizational commitment (Loi et al., 2004), and a strong attitude toward the field of work (Mael et al., 1992). Career growth encompasses the connection between a specific corporate organization and the employee’s value pursuit, which emphasizes the corporate recognition of the employee’s knowledge and work value, as well as the employee’s recognition of the company’s development prospects, including job promotion, competence, and salary level improvement. Therefore, it is reasonable to believe that employees’ career growth for the work they do is the result of a combination of self-recognition and cooperation recognition. A high level of career growth orientation creates a greater degree of organizational commitment and job satisfaction among employees, who are also more likely to use their spare time to work to obtain faster career growth.

Highly challenging work is accompanied by the high demands of the work tasks or attributes beyond the essential work routine requirements, in which employees often have a certain degree of work autonomy to decide the delivery of work results and the division of work interfaces, that is, the attitude of the implementation of work tasks from “I am required to do” to “I want to do it.” The key to both of these work attitude shifts is motivation to work; highly challenging work tasks stimulate employees’ self-efficacy, which is often linked to motivation to pursue personal goal attainment (Newstrom, 2015), so employees are more likely to be self-driven and proactive in completing work delivery efficiently when goals are clear, well-defined, progress and content are clearly controllable.

Employees who seek high levels of job competency and job proficiency are more willing to take on highly challenging tasks, employees with this trait are generally less likely to experience role overload and role ambiguity (Biron and Bamberger, 2010). It is also well established that effective control over work tasks increases employees’ organizational commitment and enhances their responsibility (Wrzesniewski and Dutton, 2001), thus high challenges are a motivating factor in terms of promoting employees’ work autonomy and improving the quality of outcome delivery. Then, the ability of higher job challenges to promote employees to initiate overtime work is effectively explained.

Finally, workload might directly lead to increased work stress, which will affect employees both physically and psychologically, resulting in reduced work effectiveness, work quality, and health status (Gilboa et al., 2008; Luchman and Gonzalez-Morales, 2013), and may make it difficult to meet family obligations due to high workload and leave the workforce (Coverman, 1989; Harris and Harris, 2008). The key source of workload is an excessive amount of work tasks, when faced with a task requirement that exceeds employees’ capability, these requirements may break through the limits of their ability and mental tolerance range, which in turn produces role ambiguity and role overload, and this work task will further produce work overload when there is only less time to complete it.

## 5. Conclusion and insights

### 5.1. Research findings

Based on the background of the two-factor theory, this study categorizes the factors that lead to overtime work of employees in enterprises as motivational factors and hygiene factors, questionnaire design based on proven test scales, uses factor analysis and data modeling to conduct empirical analysis, draws empirical conclusions and supports the results. The core conclusions include the following three main points: First, both motivational and hygiene factors positively promote employees’ active overtime work. Overtime culture, institutional system, and well-designed physical office environment are all possible to positively promote active overtime motivation. Factors of the job itself such as workload can positively promote employees’ overtime behavior and frequency but play a negative role in promoting the degree of willingness to actively work overtime.

Second, the promotion of overtime by each dimension of overtime behavior shows a “limited” pattern. From the demographic point of view, male employees as a whole show a higher tendency to work overtime initiatively, they have better adaptation and compliance to overtime corporate culture, and are more likely to be influenced by marital status; employees’ willingness to work overtime increases gradually with age; employees with higher education level also attach relatively higher importance to the physical work environment and are more likely to work overtime due to the workplace environment and atmosphere. In addition, employees with higher levels of education are also more likely to work overtime initiatively for higher personal career development, higher financial rewards, and high intensity and challenging work content; the level of actual income has a smaller effect on the motivation to work overtime relative to the level of expenditure, and employees who spend a higher percentage of their income are more likely to volunteer to work overtime.

Finally, work planning and personal habits can affect the willingness to work overtime voluntarily. A clear career plan can promote employees to work overtime actively to a certain extent, and they are more likely to work overtime actively due to the physical office environment, job growth, economic rewards, workload, and work challenges, but employees with a clear career plan are less influenced by system-oriented overtime, and even play a reverse role in promoting it; the high relevance of the work performed and the profession will also form a positive role in encouraging employees to work overtime actively. Employees at higher position levels are more motivated to work overtime due to the institutional environment and physical environment but are less influenced by the overtime-based corporate culture and are more financially rewarded. A high level of work control and self-determination can promote employees’ active adaptation to overtime culture, which in turn positively influences their willingness to work overtime voluntarily due to a good physical office environment. Moreover, daily overtime behavior does not form employees’ subjective adaptation to overtime culture, or even plays the opposite role, but the high frequency and high intensity of overtime will have a certain promotion effect on employees’ self-identification within the overtime system.

### 5.2. Research innovation

This study innovatively applies the two-factor theory to the analysis of overtime motivation, dividing the factors that lead to overtime into organizational environment factors and work itself factors. In addition, this study analyzes the differences in overtime motivation among different group of people, which serves as a supplement and extension of the two-factor theory and exploration of organizational management and talent motivation.

In terms of research methodology, we creatively applied the scale based on the factors that have been proven to influence employees’ overtime work to this study. To ensure that the test scale has relatively high reliability and sterilization, we used a combination of exploratory factor analysis and validation factor analysis research methods. In addition, based on the results of the factor analysis study, the empirical research method of data modeling was used to explore the real influencing factors that promote employees’ initiative to work overtime. In terms of research findings, this study also verifies that hygiene factors also have positive motivational effects in terms of employee-initiated overtime work, which provides some reference for employee motivation and job design.

### 5.3. Research insights

Based on the hypothesis that proactive overtime is an important manifestation of high motivational work design, this study argues that effective work design is an important and core part of high-performance work systems, and that job function design and work environment design are key components of this system. Based on the results of this study, we found that in the overtime dimension, some of the conditions with the characteristics of hygiene factors are also motivating, and some of the factors with motivating effects are negatively motivating. Based on this, this study proposes the following insights from the motivational perspective of work environment and work content design.

From the perspective of the work environment dimension, the influence of corporate culture on employees’ performance behavior cannot be ignored. A well-oriented corporate culture can motivate employees’ behavior, and a culture that employees identify with can play a strong motivational role. Secondly, institutional construction as a potential factor that can improve employee motivation cannot be ignored. It is generally believed that corporate management includes institutional management and cultural management stages, and institutional management is considered an active restraint. For this study, institutional conventions with high clarity, transparency, and clear orientation also have certain motivational effects, forming employees’ subjective compliance with corporate rules and regulations, mainly manifested by male employees. Employees with highly relevant career plans are more likely to be oriented by the system and show. Female, highly educated, married (child-bearing) employees are more likely to work overtime voluntarily because of the well-designed and comfortable office environment.

In terms of the work itself, good job design can also be motivating, but this motivational effect needs to be based on a certain foundation. Male employees, compared with female employees, pay more attention to career growth, job challenges, and financial rewards, and are more likely to work overtime voluntarily because of oriented, high reward, and high growth jobs. Second, we should pay attention to career planning and education levels, as higher education levels are highly correlated with higher career planning and highly educated employees are more likely to be influenced by career growth, financial rewards, and job challenges. Furthermore, focusing on the scope of the application of financial incentives, linking financial rewards to work overtime does not increase employees’ motivation to do overtime work.

### 5.4. Research limitations

The measurement scales used in this study were adapted, retested, and applied from scales that have been shown to cause overload and workaholism. Although they have relatively good reliability and validity in this study, the data were collected in China, and their applicability in other countries around the world is yet to be verified due to the current situation, demographics, and political environment in China.

We mainly used a cross-sectional study approach in the conduct of this study, which has major flaws in causal analysis and makes it difficult to derive on very accurate causal relationships. However, this does not mean that the results of the study are distorted or meaningless, as Theorell and Hasselhorn (2005) point out that cross-sectional analysis studies help to identify potential risks and problems. The innovative application of two-factor theory in academic research exploring overtime motivation is just such an attempt that has practical value. Moreover, we did not collect data on actual attendance in each company, and the data included in the analysis study were all from the supervisors’ feelings of the researched subjects, so there is a relatively large deficiency in the data depth research exploration.

We conducted the research and collected data during the Covid-19 pandemic in China, Respondents may be influenced by the prevailing social and economic environment and the form of corporate development in China, especially when the company’s existing business is unsustainable due to the Covid-19, employees may be more negative and conservative subconsciously, worrying about the risk of unemployment, decreasing expectations for the future, and decreasing economic returns, etc. Therefore, it is not known whether the results obtained from the research data are still true and valid in the context of the new crown.

## Data availability statement

The original contributions presented in the study are included in the article/Supplementary material, further inquiries can be directed to the corresponding author.

## Author contributions

JT: conceptualization, validation, resources, and writing—review and editing. CZ: methodology, formal analysis, and writing—original draft preparation. CZ and ZL: data curation. ZL: manuscript editing. All authors contributed to the article and approved the submitted version.

## Funding

This research was supported by the XJP Center of East China University of Political Science and Law under grant number 2021XJP07.

## Conflict of interest

The authors declare that the research was conducted in the absence of any commercial or financial relationships that could be construed as a potential conflict of interest.

## Publisher’s note

All claims expressed in this article are solely those of the authors and do not necessarily represent those of their affiliated organizations, or those of the publisher, the editors and the reviewers. Any product that may be evaluated in this article, or claim that may be made by its manufacturer, is not guaranteed or endorsed by the publisher.
