# Supplementary material for: Why do employees actively work overtime? The motivation of employees’ active overtime in China
Source: Front Psychol. 2023 Apr 24;14:1120758. doi: 10.3389/fpsyg.2023.1120758 (PMC10166067; doi:10.3389/fpsyg.2023.1120758)
Supplement: Supplementary file 1 [file Data_Sheet_1.docx]

**Appendix**

**Questionnaire**

**Questionnaire Research on Overtime Work of Employees**

Hello,

Thank you very much for taking your valuable time to fill out this questionnaire, which is designed to investigate the current situation and motivation of overtime work of contemporary enterprise workers and is filled out anonymously for academic research purposes only and for no other purpose, so please feel free to fill it out.

**Part：A**

This part is a summary of basic information, and is filled out anonymously, so please feel free to fill it out. (Please put a tick in front of "□" for the option that best matches)

A1. Your gender?

□male □female

A2. Your age?

□Under 20 years old □21-25 years old □26-30 years old □31-35 years old □36-40 years old □41-45 years old □46-50 years old □51-55 years old □55 years old and above

A3. Your education?

□Specialist and below □Bachelor's degree □Master's degree □Doctoral degree

A4. Your marital status?

□Unmarried □Married with no children □Married with children

A5. Your average monthly income before tax (including salary, bonus, etc., excluding investment income)?

□Under 6000 RMB □6000-10000RMB □10000-150000RMB □15000-20000RMB

□20000-30000RMB □30000-50000RMB □50000RMB and above

A6. What is your average monthly living expense as a proportion of your income? (Expense including rent, mortgage, car loan, medical care, buying household goods, children's education, supporting the elderly, etc.)

□under 20% □20%-40% □40%-60% □60%-80% □80% and above

A7. The number of employees in your company?(Enterprise Size)

□Less than 100 people □100-300 people □300-1000 people

□1000-2000 people □2000 people and above

A8. Category of your job?

□Technology/R&D □Management/Administration □Marketing

□Teachers/Consultants/Consultants □Professional (such as accountants, lawyers, architects, medical professionals, journalists, etc.)

□Skilled workers □Other ____

A9. Your position level?

□General Staff □Junior Manager (supervisor, manager and related position) □ Middle Manager (directors and related positions) □Senior Managers

A10. How relevant is your current job to your career plan?

□Not at all relevant □Not relevant □Partially relevant

□Very relevant □Completely relevant

A11. How relevant is your current job to your field of expertise/study?

□Not at all relevant □Not relevant □Partially relevant

□Very relevant □Completely relevant

**Part:B**

This section is mainly related to the current situation and motivational factors of overtime work in the work process. (Please put a tick in front of "□" for the option that best matches)

B1. How much of the workload (or intensity of work) is determined by yourself in your actual job?

□Completely unable to decide □Partially unable to decide □Uncertain □Mostly able to decide on their own □Completely able to decide on their own

B2. What is your average frequency of overtime work per week in the past six months?

□less than 1 time □2-3 times □4-5 times □more than 5 times

B3. What is the average length of time you work each overtime shift?

□0-1 hour □2-3 hours □4-5 hours □6-8 hours □8 hours or more

B4. To what extent do you voluntarily work overtime?

□Completely involuntary □Involuntary □Not exclusive nor pandering □Voluntary □Completely voluntary

B5. How likely are you to work overtime due to the following scenarios and conditions, with "1" indicating "very unlikely" and "5" indicating "very likely"?

Part Ⅰ: Actual overtime work due to work environment

| **Title item** | **Degree** | **Rating from "1" to "5", the higher the score, the higher the likelihood** | | | | |
| --- | --- | --- | --- | --- | --- | --- |
| / | / | 1 | 2 | 3 | 4 | 5 |
| Ⅰa | In keeping with the overtime behavior of executives | □ | □ | □ | □ | □ |
| Ⅰb | To align with the overtime behavior of direct supervisors | □ | □ | □ | □ | □ |
| Ⅰc | To keep in line with the overtime behavior of colleagues | □ | □ | □ | □ | □ |
| Ⅰd | In order to be consistent with the overall overtime behavior of the company | □ | □ | □ | □ | □ |
| Ⅰe | The company provides a safe and comfortable office environment | □ | □ | □ | □ | □ |
| Ⅰf | The company provides resources required for overtime work, such as overtime meals, rest and fitness areas | □ | □ | □ | □ | □ |
| Ⅰg | The company provides resources to facilitate goal achievement | □ | □ | □ | □ | □ |
| Ⅰh | Company system promotes overtime work | □ | □ | □ | □ | □ |
| Ⅰi | Overtime hours can be redeemed for related benefits | □ | □ | □ | □ | □ |
| Ⅰj | The system agrees that overtime work will be directly or indirectly applied to promotions, pay raises, performance, etc. | □ | □ | □ | □ | □ |

Part Ⅱ: Actual overtime work caused by the work itself

| **Title item** | **Degree** | **Rating from "1" to "5", the higher the score, the higher the likelihood** | | | | |
| --- | --- | --- | --- | --- | --- | --- |
| / | / | 1 | 2 | 3 | 4 | 5 |
| Ⅱa | Working overtime to acquire more knowledge in your field of work | □ | □ | □ | □ | □ |
| Ⅱb | Working overtime for - to get a promotion | □ | □ | □ | □ | □ |
| Ⅱc | Working overtime to achieve career aspirations faster | □ | □ | □ | □ | □ |
| Ⅱd | To get a higher salary income | □ | □ | □ | □ | □ |
| Ⅱe | To receive direct financial benefits, such as overtime pay | □ | □ | □ | □ | □ |
| Ⅱf | To gain potential benefits from overtime, such as increased base pay, extra incentives, high performance, etc. | □ | □ | □ | □ | □ |
| Ⅱg | Working overtime due to excessive work tasks | □ | □ | □ | □ | □ |
| Ⅱh | Work overtime because of the difficulty and high demand of the task you are responsible for | □ | □ | □ | □ | □ |
| Ⅱi | Working overtime because of lack of time | □ | □ | □ | □ | □ |
